# Regulation of RAGE for Attenuating Progression of Diabetic Vascular Complications

**DOI:** 10.1155/2012/894605

**Published:** 2011-10-29

**Authors:** Myat Thu Thu Win, Yasuhiko Yamamoto, Seiichi Munesue, Hidehito Saito, Dong Han, So Motoyoshi, Tarek Kamal, Takuro Ohara, Takuo Watanabe, Hiroshi Yamamoto

**Affiliations:** Department of Biochemistry and Molecular Vascular Biology, Kanazawa University Graduate School of Medical Science, Kanazawa 920-8640, Japan

## Abstract

Diabetic angiopathy including micro- and macroangiopathy is concerned with high rate of morbidity and mortality in patients with long-standing diabetes. Receptor for advanced glycation end products (RAGE) and its ligands have been considered as important pathogenic triggers for the progression of the vascular injuries in diabetes. The deleterious link between RAGE and diabetic angiopathy has been demonstrated in animal studies. Preventive and therapeutic strategies focusing on RAGE and its ligand axis may be of great importance in relieving diabetic vascular complications and reducing the burden of disease.

## 1. Introduction

Diabetes is a life-threatening disease attributing to its devastating complications such as cardiovascular disease, stroke, and microvascular diseases. The population worldwide with diabetes is estimated to be 285 million adults in 2010, and it will reach 439 million adults by 2030 with overall total predicted 54% increase [1]. The very recent study reports the number of people with diabetes already came to 347 million in 2008 [2]. In proportion to the rapid increase of diabetic population, diabetic nephropathy is now a major cause of end-stage renal disease and diabetic retinopathy is a leading cause of blindness [3]. Extensive intracellular and extracellular formation of advanced glycation end-products (AGE) is considered to be a causative factor in sustained hyperglycemia-induced vascular injuries in diabetes. Receptor-dependent and receptor independent mechanisms are known to work in the AGE-induced cellular dysfunction and tissue damage. The receptor for AGE (RAGE) is originally found as an AGE-binding receptor and now recognized as a proinflammatory molecular devise mediating danger signals to the body. In this paper, our current understanding about RAGE and its multiligand system will be reviewed remarkably in the development and progression of diabetic vascular complications and in possible therapeutic targets for these diseases.

## 2. AGE

Maillard first described the formation of brown-colored substances resulted from nonenzymatic reaction between reducing sugar and proteins [4]. There is a chemical linkage between the carbonyl groups and the amino group to form Schiff bases and then Amadori compounds, followed by irreversible dehydration, condensation, and crosslinking, resulting in heterogeneous derivatives termed AGE [5]. Similar reactions have been found to occur with nonglucose materials containing an aldehyde group by enzymatic and non-enzymatic pathways. Metabolites from glycolysis pathway such as dicarbonyls of methylglyoxal (MG), glyoxal, and 3-deoxyglucosone (3DG) are known to interact with protein residues to rapidly form AGE [6]. Reactive dicarbonyls can be also generated from ketones, lipids, and other metabolic pathways [7]. Increased production of the reactive dicarbonyls or reducing detoxification by the glyoxalase system or endogenous scavengers leads to a state of carbonyl stress, which is increasingly considered to be the major driving force for AGE formation and accumulation [8]. Glyoxal is also produced by auto-oxidation of glucose and 3-deoxyglucosone (3DG) generated by hydrolysis from Amadori rearrangement products [9]. Glycolaldehyde production by myeloperoxidase from activated macrophages and neutrophils plays a pathogenic role in generating AGE and damaging tissues at sites of inflammation [10]. Formation of AGE is accelerated under hyperglycemia in diabetes. Some AGE have intrinsic fluorescence which can be used as a surrogate marker for the presence of AGE modifications. AGE are chemically heterogeneous groups of compounds with only 25 AGE structures fully characterized. N^*ε*^-carboxymethyl-lysine (CML) is the simplest and best characterized AGE and the main epitope for recognition by most commercially available antibodies used for the detection and quantification of AGE. Exogenously offered AGE are absorbed in the gastrointestinal tract (~10%), and approximately two-thirds remained in contact with tissues for >72 hr, whereas the rest is rapidly excreted by the kidneys [11–13]. Reducing basal oxidative stress by AGE restriction in mice, without energy or nutrient change, alleviates inflammation, prevents vascular complications, and extends normal life span [14, 15].

## 3. RAGE

**Figure 1:**
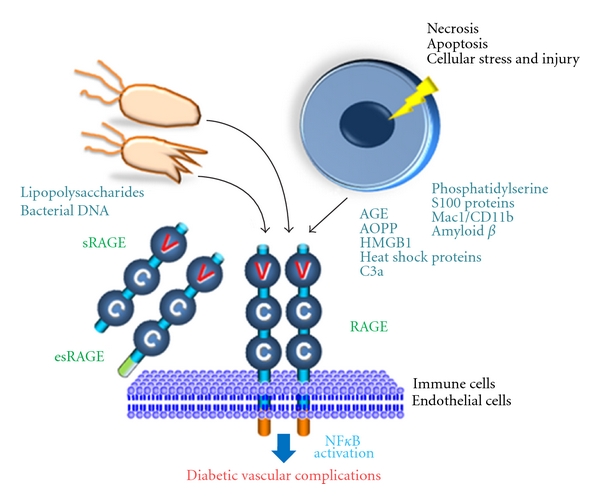
RAGE and its ligands play a role in the development of diabetic vascular complications. Soluble RAGE (sRAGE) and endogenous soluble RAGE (esRAGE) may work as decoy receptors against ligand-RAGE interactions. AGE: advanced glycation end product; AOPP: advanced oxidation protein product; HMGB1: high-mobility group box protein 1.

The best characterized AGE receptor is RAGE. RAGE belongs to the immunoglobulin (Ig) superfamily of cell-surface molecules and is composed of an extracellular region containing one V-type and two C-type Ig domains [16]. This portion of the receptor joins a hydrophobic transmembrane-spanning domain and then a highly charged, short cytoplasmic domain that is essential for post-RAGE signaling [16, 17]. RAGE is a member of a family of pattern-recognition receptors that function at the interface of innate and adaptive immunity. Its endogenous and exogenous binding partners include AGE, high-mobility group box 1 (HMGB1), calcium-binding S100 protein group, *β*2-integrin Mac/CD11b, amyloid *β*-peptide, *β*-sheet fibrils, advanced oxidation protein products (AOPPs), complement C3a, lipopolysaccharides (LPS), and phosphatidylserine on the surface of apoptotic cells [18–24] (Figure 1). Ligand engagement of RAGE activates the nuclear factor-*κ*B (NF-*κ*B) and other signaling pathways through stimulation of ERK (extracellular signal-regulated kinase)1/2, p38 MAPK (mitogen-activated-protein-kinase)-JNK (c-Jun N-terminal kinases), JAK (Janus-kinase)-STAT (signal transducer and activator of transcription), and Rac-Cdc42, many of which are the results as well as the cause of reactive oxygen species (ROS) [25]. Subsequently, expression of inflammatory cytokines is increased, which leads to an inflammatory response with associated cellular migration and proliferation. Recently, mammalian homologue of *Drosophila* gene *Diaphanous 1* (*mDia1*) has been identified as a direct binding partner of an intracellular domain of RAGE and as a part of the machinery of RAGE intracellular signaling [26]. The mDia1 exists widely from yeast to the mammal and is known to link with cell division, polarity formation, and movements by actin polymerization [26]. Ligation of RAGE causes a positive feed-forward loop, in which inflammatory stimuli activate NF-*κ*B, which induces RAGE expression, followed again by NF-*κ*B activation [27].

Self-downregulation system of RAGE is also reported: as an example, the binding of HMGB1 to RAGE induces an intracellular signal transduction as well as RAGE shedding by a disintegrin and metalloproteinase domain-containing protein 10 (ADAM10) [28]. The cleavage of the membrane-bound full-length and signal transducing RAGE yields soluble RAGE (sRAGE), which could work as a decoy receptor against ligand-RAGE interactions. In the strict sense of the word, sRAGE is a heterogeneous population of total sRAGE proteins, including soluble splice variants of RAGE and the proteinase-cleaved forms of membrane-bound RAGE and of the soluble splice variants [29]. Endogenous secretory RAGE (esRAGE) is one of the major splice variants of RAGE detected in blood, cell surface and cytoplasm of vascular endothelial cells, pancreatic *β*-cells, monocytes, macrophages, and so on [30, 31]. The sRAGE is thought to act locally and systemically as a decoy receptor. Reinforcing the ectodomain shedding will decrease a total amount and expression of signal-transducing RAGE and will reciprocally increase an amount of decoy receptor sRAGE; this can control ligand-RAGE signaling and subsequent cellular and tissue derangement [32]. It is also reported that sRAGE mediates inflammation by directly binding to monocytes under less ligand conditions though the mechanism of action is unknown [33]. Recent clinical studies have focused on the significance of circulating sRAGE or esRAGE in diabetic vascular complications. Findings in both type 1 and type 2 diabetic patients are quite confusing and both inverse and positive correlations have been reported in diabetic retinopathy, nephropathy, and incident cardiovascular disease events and mortality outcomes [29, 34–39]. First, production of sRAGE and esRAGE is inducible. Second, the presence of renal insufficiency can strongly and positively influence circulating sRAGE or esRAGE level [29]. Third, medications may alter sRAGE or esRAGE level. All phenomena may explain controversial findings of sRAGE or esRAGE in diabetes.

## 4. Other Receptors

Many other AGE receptors and soluble binding proteins interacting with AGE may play an important role in the AGE homeostasis: scavenger receptors class A (MSR-A), class B (MSR-B) (CD36 and LOX1), AGE-R1 (OST48 oligosaccharyltransferase), AGE-R2 (80K-H protein kinase C substrate), AGE-R3 (galectin-3), and toll-like receptor (TLR) 4 [40–44]. There are also other molecules like lysozyme and lactoferrin-like polypeptide involving in cellular uptake and degradation of AGE [45]. AGE-R1 is a type I transmembrane receptor found on the plasma membrane and in the endoplasmic reticulum [40]. The cell surface membrane-associated AGE-R1 blocks responses to AGE by blocking the induction of ROS-mediated activation of MAPK/Ras and inflammatory molecules, induced in part via RAGE [46]. Torreggiani et al. showed that the overexpression of AGE-R1 in mice is associated with decreased basal levels of circulating and tissue AGE and oxidative stress and significant protection against wire injury-induced femoral artery intimal hyperplasia and inflammation [47]. AGE-R3 (galectin-3) is also reported to work as an AGE receptor to protect AGE-induced tissue injury via AGE removal or degradation [41, 42].

## 5. Diabetic Nephropathy

To evaluate whether RAGE and ligand system may participate in the development of diabetic nephropathy, we created transgenic mice that overexpress human RAGE proteins in endothelial cells and crossbred them with another transgenic mouse line that develops insulin-dependent diabetes early after birth [48]. The resultant double transgenic mice showed significant increases in kidney weight, albuminuria, glomerulosclerosis, and serum creatinine compared with the diabetic control [48–50]. Indices diagnostic of diabetic retinopathy were also most prominent in double transgenic mice. Our group also generated RAGE knockout (KO) mice and reported the marked improvement of nephromegaly, albuminuria, glomerulosclerosis, and increase of serum creatinine level in diabetic RAGE-KO mice [51]. The kidneys of streptozotocin (STZ)-injected RAGE-KO mice were reported to be protected from early mesangial matrix expansion and thickening of the glomerular basement membrane (GBM) seen in wild-type diabetic mice [52]. Moreover, RAGE deletion was also beneficial to diabetic nephropathy seen in OVE26 type 1 diabetic mice with progressive glomerulosclerosis and decline of renal function [53]. Rüster et al. reported an interaction between the renin-angiotensin system and the AGE-RAGE axis in podocytes [54]. Since intraglomerular angiotensin II levels are increased in diabetic nephropathy, this interaction may have pathophysiological consequences for podocyte injury and inflammation associated with the development of diabetic nephropathy [54]. Pharmacological blockade of RAGE, using sRAGE in *db*/*db *diabetic mice, protected against glomerulosclerosis and other classical lesions of early diabetic nephropathy [52]. It admits no doubt that RAGE plays a major role in diabetic nephropathy (Table 1).

## 6. Diabetic Retinopathy

Diabetes retinopathy is classified into nonproliferative stage (NPDR) and proliferative stage (PDR). NPDR is characterized by capillary microangiopathy, microaneurysms, basement membrane thickening, and loss of pericytes [55]. An association between accumulation of CML and expression of vascular endothelial growth factor (VEGF) has also been found in eyes with non-PDR and PDR [56]. VEGF appears to be the most important growth factor in diabetic retinopathy. It is also well documented that oxidative stress is linked to AGE formation, and involved in retinal vascular dysfunction [57]. RAGE expression has been predominantly localized to glia in the inner retina, and the expression appeared to be upregulated in diabetic conditions [58]. Other ligands for RAGE including S100 proteins and HMGB1 are evident in the vitreous and preretinal membranes of eyes with PDR [59]. Kaji et al. demonstrated blood-retinal barrier breakdown and increased leukostasis in endothelial RAGE-overexpressing mice and their amelioration by the treatment of sRAGE [60]. RAGE activation by ligand in Müller glia results in ERK1/2 activation and subsequent production of inflammatory cytokines such as VEGF and MCP-1, implicating the critical role of RAGE in neovascularization and recruitment of immune cells such as microglia into the deep retinal layers during diabetic retinopathy to induce inflammation [58].

## 7. Diabetic Neuropathy

In diabetic neuropathy, both autonomic and peripheral nerves are affected. Endothelial injury may impair the blood flow which leads to hypoxia and oxidative stress in peripheral nerves [61]. Wada and Yagihashi demonstrated the expression of RAGE in the perineural and endoneurial endothelial cells and schwann cells of peripheral nerve in rat by *in situ *hybridization [62]. Models of experimental diabetic neuropathy provided sound evidence that deletion of the RAGE gene protected animals from the detrimental effects of diabetes, while overexpression of RAGE promotes diabetic neuropathy [63–66]. Moreover, the loss of thermal pain perception observed in mice with diabetes could be prevented by treatment with sRAGE. In agreement with these observations, NF-*κ*B activation and the loss of pain perception were largely blunted in RAGE-KO mice [63].

## 8. Atherosclerosis

AGE stimulate ROS generation in vascular wall cells and subsequently induce redox-sensitive atherosclerosis-related molecular expression such as MCP-1, matrix metalloproteinase-9 (MMP-9), and plasminogen activator inhibitor-1 (PAI-1), all of which could contribute to the formation and destabilization of coronary atherosclerotic plaques [67, 68]. AGE induce increased vascular permeability, procoagulant activity, migration of macrophages and T cells into the intima, and impairment of endothelium-dependent relaxation [69]. AGE inhibition attenuated accelerated atheroma associated with diabetes [70]. STZ-induced diabetic ApoE-KO mice clearly showed that RAGE activation has a central role in the formation and progression of atherosclerotic lesions and that the absence of RAGE was associated with a significant attenuation of the atherosclerotic plaque [71]. Competitive inhibition of RAGE by exogenously administrated sRAGE resulted in a decrease in mean atherosclerotic lesion area and number of complex lesions [72, 73]. In addition, RAGE inactivation also inhibited atherosclerosis through blocking the RAGE-mediated inflammatory reactions and oxidative stress in nondiabetic models with atherosclerosis of ApoE-KO and low-density lipoprotein (LDL) receptor KO mice [74].

In atherosclerosis, increased level of LDL and presence of small dense LDL are strong and independent risk factors and both are features of dyslipidemia [75]. The specific protein of apolipoprotein B (ApoB) on LDL is glycated in diabetes [76], and this leads to rapid scavenger uptake of the LDL [77], which gives rise to foam cell formation in atherogenesis [69]. The oxidation and glycation of LDL are partially interdependent and indisputably coexist, and both prevent LDL receptor-mediated uptake and promote macrophage scavenger receptor-mediated LDL uptake. Small dense LDL is more preferentially glycated *in vivo* and more susceptible to glycation *in vitro* than buoyant LDL [78]. Glycated-high density lipoprotein (HDL) has also been linked to decreased ability to prevent monocyte adhesion to aortic endothelial cells [79], while lipoprotein A glycation has been shown to increase PAI-1 production and decrease t-PA generation [80, 81]. Glycated LDL and HDL may also play an important role in atherogenesis.

## 9. Conclusion

Diabetic angiopathy is still a burden disease worldwide even though new pharmaceutical interventions are available. Applications of inhibitors for AGE and RAGE may be prospective therapeutic approaches for prophylaxis and treatment of diabetic angiopathy. Benfotiamine is a synthetic S-acyl derivative of thiamine and has antioxidant and anti-AGE formation properties [82]. Thiazoliumcompounds ALT-711 (algebrium), C36, TRC4186, and TRC4149 and their prototype *N*-phenacylthiazolium bromide (PTB) are known as AGE breakers, which break preaccumulated AGE or existing AGE cross-links [83–86]. 

TTP488 is an antagonist against RAGE [87]. Low-molecular-weight heparin (LMWH) can bind RAGE and act as an antagonist to RAGE [51]. LMWH treatment of mice showed preventive and therapeutic effects on albuminuria and increased glomerular cell number, mesangial expansion, and advanced glomerulosclerosis in a dose-dependent manner [51]. Thiazolidinediones, calcium channel blockers, angiotensin-converting enzyme inhibitors (ACEIs), angiotensin II receptor blockers (ARBs), and statins are reported to suppress RAGE expression [88–90]. Treatment of statins and ACEI stimulated circulating sRAGE production in human studies [91, 92]. It is interesting in the future to develop new devices and remedies of controlling the RAGE ectodomain shedding. New therapeutic strategies are desired to prevent diabetic vascular complications and to improve both quantity and quality of life in patients with diabetes.

## Figures and Tables

**Table 1:** RAGE and its ligand axis in diabetic angiopathy using RAGE gene-manipulated animal models.

Animal models	Phenotypes	References
*Diabetic nephropathy*		
RAGE transgenic mice	Nephropathy ↑	[48, 50]
(Type 1 diabetes)		
+ AGE inhibitor	Nephropathy ↓	
		
RAGE knockout mice	Nephropathy ↓	[51, 93]
(Type 1 diabetes)		
+ LMWH treatment	Nephropathy ↓	
RAGE knockout mice	Nephropathy ↓	[53]
(Type 1 diabetes, OVE26)		
*db/db* mice + sRAGE	Nephropathy ↓	[52]
RAGE knockout mice	Nephropathy ↓	
(STZ-diabetes)		

*Diabetic retinopathy*		
RAGE transgenic mice	Retinopathy ↑	[60]
(STZ-diabetes)		
+ sRAGE treatment	Retinopathy ↓	
RAGE transgenic mice	Retinopathy ↑	[80]
(Type 1 diabetes)		

*Diabetic neuropathy*		
RAGE knockout mice	Neuropathy ↓	[63]
(STZ diabetes)		
+ sRAGE treatment	Neuropathy ↓	
RAGE knockout mice	Neuropathy ↓	[64]
(STZ diabetes)		
